# Preoperative opioid use is associated with worse patient outcomes after Total joint arthroplasty: a systematic review and meta-analysis

**DOI:** 10.1186/s12891-019-2619-8

**Published:** 2019-05-18

**Authors:** C. Michael Goplen, Wesley Verbeek, Sung Hyun Kang, C. Allyson Jones, Donald C. Voaklander, Thomas A. Churchill, Lauren A. Beaupre

**Affiliations:** 1 Department of Surgery, University of Alberta, Edmonton, AB T6G 2B7 Canada; 2 Faculty of Medicine and Dentistry, University of Alberta, Edmonton, AB T6G 2R7 Canada; 3 Alberta Bone and Joint Institute, Calgary, Alberta T2N 4Z6 Canada; 4 Department of Physical Therapy, University of Alberta, Edmonton, AB T6G 2G4 Canada; 5 School of Public Health, University of Alberta, Edmonton, Alberta T6G 1C9 Canada

**Keywords:** Opioids, Total knee arthroplasty, Total hip arthroplasty, Patient-reported outcomes

## Abstract

**Background:**

A significant number of patients use opioids prior to total joint arthroplasty (TJA) in North America and there is growing concern that preoperative opioid use negatively impacts postoperative patient outcomes after surgery. This systematic review and meta-analysis evaluated the current evidence investigating the influence of preoperative opioid use on postoperative patient-reported outcomes (PRO) after total joint arthroplasty.

**Methods:**

A systematic search was performed using Ovid, Embase, Cochrane Library, Scopus, Web of Science Core Collection, CINAHL on February 15th, 2018. Studies reporting baseline and postoperative PRO among those prescribed preoperative opioids and those who were not prior to total knee and hip arthroplasty were included. Standardized mean differences (SMD) in absolute difference and relative change in PRO measures between the two groups was calculated using random effect models.

**Results:**

Six studies were included (*n* = 7356 patients); overall 24% of patients were prescribed preoperative opioids. Patients with preoperative opioid use had worse absolute postoperative PRO scores when compared to those with no preoperative opioid use (standardized mean difference (SMD) -0.53, 95% Confidence interval (CI) -0.75, − 0.32, *p* < 0.0001). When relative change in PRO score was analyzed, as measured by difference between postoperative and preoperative PRO scores, there was no group differences (SMD -0.26, 95% CI -0.56, 0.05, *p* = 0.10).

**Conclusion:**

Patients prescribed preoperative opioids may attain worse overall pain and function benefits after TJA when compared to opioid-naïve patients, but do still benefit from undergoing TJA. These results suggest preoperative opioid users should be judiciously counselled regarding potential postoperative pain and function improvements after TJA.

**Electronic supplementary material:**

The online version of this article (10.1186/s12891-019-2619-8) contains supplementary material, which is available to authorized users.

## Background

Over the past 20 years, the number of opioids prescribed to manage patients with chronic non-cancer pain, such as arthritis has dramatically increased in North America [1, 2]. The reported rise is thought to be related to American guidelines that supported opioids to manage pain associated with arthritis [3]. Unfortunately, these guidelines were largely based on expert opinion and industry-backed studies with little supporting evidence [4, 5]. Emerging evidence now suggests that opioids provide no benefit when compared to ibuprofen or acetaminophen to manage pain associated with arthritis, but had higher rates of adverse events [6, 7]. Nevertheless, physician prescribing practices have resulted in over 40% of patients being prescribed opioids prior to total joint arthroplasty (TJA) in the USA [8–11].

Opioid use prior to TJA use has gained significant clinical and research interest given its potential to prognosticate a patient’s postoperative outcome [8, 9, 12, 13]. Preoperative opioid use has been associated with a more complicated hospital course and more complications after TJA. Sing et al. (2016) reported that preoperative opioid users, stayed on average 1.6 days longer in hospital (*p* = 0.05), were more likely to be discharged to a subacute facility (OR 6.7, 95% CI 2.4, 19.0) and associated with increased 90-day complications rates (OR 6.2, 95% CI 1.5, 26.0) than those who did not use opioids preoperatively [12]. Further, Ben-Ari et al. (2017) reported on 32,636 patients who underwent total knee arthroplasty (TKA), of which 39% were using long-term opioids preoperatively [9]. Patients who underwent revision surgery within 1 year were more likely to be taking opioids preoperatively, after controlling for other factors (1.4 OR, 95% CI 1.2, 1.6) [9]. However, reports are conflicting regarding the extent that preoperative opioid use impacts postoperative patient-reported outcomes (PRO) after surgery [10, 14, 15].

The primary objective of this systematic review was to investigate the impact of preoperative opioid use on PRO’s after TJA. Our secondary objectives were to: 1) determine the prevalence of preoperative opioid use and dose prior to TJA; 2) compare the parameters used to define preoperative opioid use, such as duration and dose among studies; 3) compare postoperative opioid use between those who were prescribed preoperative opioids and opioid-naïve patients; 4) describe differences in preoperative patient characteristics and postoperative discharge characteristics.

## Methods

This systematic review and meta-analysis was performed in accordance with the Preferred Reporting Items for Systematic Reviews and Meta-Analysis (PRISMA) guidelines [16].

### Search strategy

The search strategies were developed by a health research librarian in collaboration with the first author (CG) and the following databases were searched on February 15th, 2018: 1) Ovid MEDLINE(R) Epub Ahead of Print, In-Process & Other Non-Indexed Citations, Ovid MEDLINE(R) Daily and Ovid MEDLINE(R); 2) Embase; 3) Cochrane Library; 4) Scopus; 5) Web of Science Core Collection; 6) CINAHL Plus with Full-Text. Controlled vocabulary and text-word terms representing arthroplasty were combined with terms representing opiates/opioids and terms representing the preoperative period. No date or language limits were applied. See Additional file 1: Appendix A for the complete search strategy.

### Inclusion and exclusion criteria

Peer-reviewed articles that met the following criteria were included in our review: 1) included patients who had undergone primary total hip or total knee arthroplasty; 2) reported disease or joint specific preoperative and postoperative PRO measures; 3) compared patients prescribed preoperative opioids (hereafter ‘opioid users’) to those who were not (hereafter ‘opioid–naïve’); 4) written in English. All study designs eligible for inclusion except case reports and conference abstracts.

### Primary outcome

The primary outcome of this review was the differences in absolute postoperative PRO scores as well as relative change in PRO scores for opioid users when compared to opioid-naïve patients. Relative change in PRO score was calculated by determining the difference between preoperative and postoperative PRO score.

### Secondary outcomes

Our secondary outcomes were: 1) the prevalence of preoperative opioid use; 2) the parameters used to define preoperative opioid use, such as dose and duration; 3) postoperative opioid rates for those prescribed preoperative opioids and opioid-naïve patients; 4) postoperative health services utilization.

### Data extraction and synthesis

One investigator (CG) imported all retrieved studies into RefWorks, a reference management software program and screened titles to remove duplicate studies. All remaining studies were imported into Covidence, a screening and data extraction tool, for abstract screening, full text review and data extraction [17]. Two reviewers (CG and WV) independently screened all abstracts, completed full-text review of potentially eligible studies and extracted data from included studies. Data extracted included study design, publication date, sample size, statistical methods, preoperative patient data including age, sex and comorbidities, opioid use case definition, the prevalence of preoperative opioid use, PRO measures and secondary outcomes. Secondary outcomes included the prevalence of opioid use before and after TJA, patient demographic information for each group and healthcare utilization information including length of stay and discharge characteristics. Each reviewer then cross-checked all data and any disagreements between reviewers were discussed and resolved by consensus; no third party was required to achieve consensus. If available data were not directly extractable, the original authors were contacted (Additional file 2: Table S1).

### Statistical analysis

#### PRO scores

All extracted PRO scores and standard deviation (SD) were standardized to 100 and reversed if required so that a score of 100 indicated the best possible score. If available, total PRO score was used for all calculations, otherwise the pain scores were used. Change in PRO score for each study was calculated by calculating the difference between mean postoperative PRO score and mean preoperative PRO score for opioid users and opioid-naïve groups. The differences between groups were determined by calculating the difference between mean change in PRO score or absolute postoperative PRO score for each study. For studies reporting a mean and 95%CI, we used the formula CI = mean ± t x (SD / √n) to calculate the SD [18]. Change in score SD (S_diff_) was determined using the formula: ${S}_{\mathrm{diff}}=\sqrt{S_1^2+{S}_2^2-2\times \mathrm{r}\times {S}_1\times {S}_2\ }$, where S_1_ equals the groups mean preoperative PRO score SD, S_2_ equals the group’s postoperative score SD and r is the correlation between preoperative and postoperative scores [18]. If there was no prior information on the correlation coefficient (r), we used a value of 0.5. Our sensitivity analysis was robust when we compared the results with correlation coefficients varying from 0.3 (low) to 0.8 (high), so we used the mid-point of 0.5 for our main analysis. For the studies where the SD was not reported, the standard SD was calculated by converting the *p*-value to a t-score and solving for SD using the study sample size [18]. SMD was then calculated by entering either absolute mean PRO score or change in mean PRO score for each group into Review Manager 5.3 [19]. SMD enables continuous outcome scores that measure the same construct with different instruments to be pooled by expressing the intervention effect relative to SD rather than the original units of measurement [20]. Random effect models were used to compute pooled SMD and 95% CIs. Random-effects models account for between study heterogeneity and provides a more conservative evaluation of the association than one based on fixed effects [18]. Interpretations of effect sizes were based on suggestions by Cohen where an effect size of 0.2 is small, 0.5 is medium and 0.8 is large [21, 22]. Heterogeneity was assessed with the I^2^ statistic and interpreted as low (> 25%), moderate (> 50%), or high (> 75%) [23]. The level of significance was set at *p* < .05.

#### Prevalence of opioid use prior to TJA

The prevalence of preoperative opioid use was calculated by pooling the total number of patients prescribed preoperative opioids divided by the total number of patients in the studies that reported preoperative opioid use (*n* = 3 studies).

### Assessment of study quality

Two reviewers (CG and WV) independently conducted a quality assessment of eligible studies using the Joanna Briggs Institute (JBI) Critical Appraisal Checklist for Cohort Studies (Additional file 1: Appendix B) [24]. This checklist contains 11 questions that assess specific domains of studies to determine the potential risk of bias and could be answered with ‘yes’, ‘no’ or ‘unclear’ (Additional file 1: Appendix B). Any disagreements between reviewers were discussed and resolved by consensus. The risk of bias of individual studies were determined with the following cutoffs: low risk of bias if 70% of answers scored yes, moderate risk if 50 to 69% questions scored yes and high risk of bias if yes scores were below 50% [25, 26].

## Results

### Study selection

**Fig. 1:**
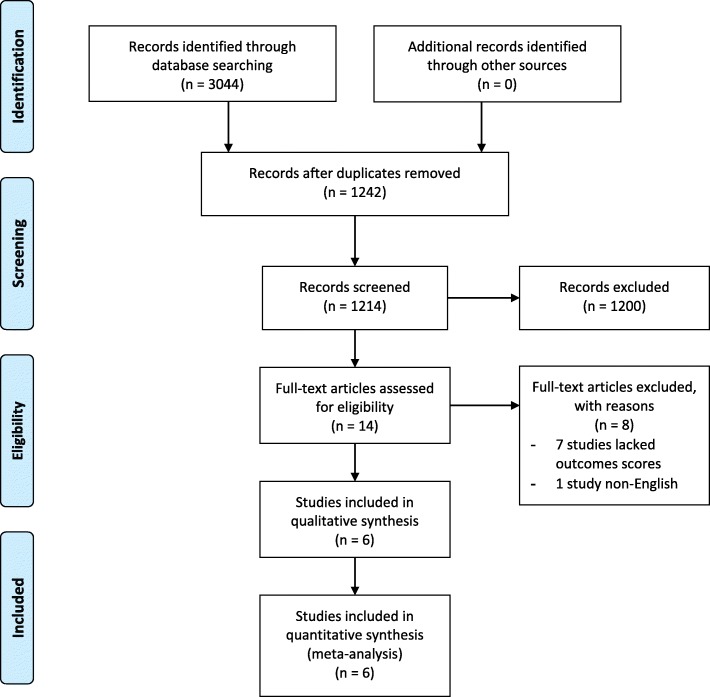
– PRISMA flow diagram

Of the 3044 studies identified from the primary search, 1830 studies were duplicates and removed, leaving 1214 studies to undergo abstract screening. After removing 1200 irrelevant studies, 14 studies were reviewed in full to determine potential eligibility for inclusion and 6 studies were included in our meta-analysis (7356 patients) [10, 27–31]. The summary of study selection is presented within the PRISMA diagram (Fig. 1).

### Study characteristics

All studies were retrospective cohort studies, conducted in the USA and published between 2010 and 2017 (Table 1). Five studies were a retrospective analysis of prospectively collected data while one study did not indicate specific details regarding source patient data (Table 1). Potentially confounding factors were controlled by using a matched cohort (*n* = 3 studies), or risk adjustment (*n* = 1 study); two studies did not control for other potentially confounding variables (Table 1). Three studies included only TKA patients, two studies combined both total hip arthroplasty (THA) and TKA patients, while one study was limited to THA patients (Table 1). The Western Ontario and McMaster Universities Osteoarthritis Index (WOMAC) was reported for three studies, two studies reported the Knee Society Score (KSS) and one study used the Harris Hip Score (HHS) (Table 1). Mean postoperative follow-up ranged from 6 months to 58 months (Table 1).

**Table 1:** Characteristics of included studies

Study	Year	Country	n	Procedure	Study Design	Source of Patients	Control of Confounding	Factors Matched/Adjusted	PRO	Follow up
Zywiel et al.	2011	USA	90	TKA	Retrospective Cohort	Prospectively collected database at two institutions that specialize in lower extremity total joint arthroplasty	Matching	Center (exact match), procedure type (unilateral or bilateral procedure; exact match), sex (exact match), age (± 4 years) and BMI (± 4 kg/m^2^)	KSS	38.5 months (mean)
Smith et al.	2017	USA	156	TKA	Retrospective Cohort	Secondary analysis of a randomized controlled trial evaluating motivational interviewing to enhance TKA outcomes	Risk Adjustment	Propensity Score (Pain Catastrophizing Scale score, Charlson Comorbidity Index and baseline WOMAC pain score), preoperative opioid use	WOMAC	6 months
Franklin et al.	2010	USA	6346	TKA	Retrospective Cohort	Prospectively data on a national sample of primary, unilateral TKA patients sponsored by Zimmer, Inc., Warsaw between 2000 and 2005	None	None	KSS	12 months
Pivec et al.	2014	USA	108	THA	Retrospective Cohort	Prospectively collected database at two institutions that specialize in lower extremity total joint arthroplasty	Matching	Gender, Unilateral or bilateral total hip arthroplasty (exact), Age (±5 years), BMI (± 4 kg/m^2^), when possible: insurance type, tobacco use ≥0.5 packs per day, history of psychiatric disorders, history of back pain or surgery	HSS	58 months (mean)
Nguyen et al.	2016	USA	82	TKA, THA	Retrospective Cohort	A single institution database	Matching	Primary diagnosis, affected joint (hip/knee), American Society of Anesthesiologists’ classification of physical health, sex, BMI (±10 kg/m^2^), age (±10), daily morphine equivalent group	WOMAC	6–12 months
Goesling et al.	2016	USA	574	TKA, THA	Retrospective Cohort	Secondary analysis of data from a prospective outcome study in patients undergoing TKA and THA	None	None ^a^	WOMAC	6 months

Abbreviations

*n* number of patients included from study, *PRO* Patient-Reported Outcome, *WOMAC* The Western Ontario and McMaster Universities Osteoarthritis Index, *KSS* Knee Society Score, *HHS* Harris Hip Score, *TKA* Total Knee Arthroplasty, *THA* Total Hip Arthroplasty, *BMI* Body Mass Index

Notes

^a^Additional data provided that did not adjust for other patient factors

### Risk of Bias

Three studies were considered to have a moderate risk of bias, while the remaining 3 studies were classified as high risk of bias according to the JBI Critical Appraisal Checklist for Cohort Studies (Table 2). Most studies lacked appropriate statistical methods or design to identify and control for differences noted between the two groups (Table 2).

**Table 2:** JBI risk of bias quality assessment for cohort studies

Study	Q1^a^	Q2	Q3	Q4	Q5	Q6	Q7	Q8	Q9	Q10	Q11	% yes	Risk^b^
Zywiel et al.	✕	✓	✓	✓	✕	✓	✓	✓	?	✕	✕	55%	Moderate
Smith et al.	✕	✕	✕	✓	✓	✓	✓	✓	?	✕	✓	55%	Moderate
Franklin et al.	?	✕	✕	✕	✕	✓	✓	✓	✕	✕	✕	27%	High
Pivec et al.	✕	✓	✓	✓	?	✓	✓	✓	?	✕	✕	55%	Moderate
Nguyen et al.	?	✓	✓	✕	✕	✓	✓	✓	?	✕	✕	45%	High
Goesling et al.	✕	✓	✕	✓	✕	✓	✓	✓	?	✕	✕	45%	High

Abbreviations

*JBI* Joanna Briggs Institute

^a^Q1 – Q11 indicate questions 1 to 11 based on the JBI risk assessment (Additional file 1: Appendix B).

Notes

^b^The risk of bias was ranked as high when the study reached up to 49% of “yes” scores, moderate when the study reached from 50 to 69% of “yes” scores, and low when the study reached more than 70% of “yes” scores. ‘✓’ indicates yes, ‘✕’ indicates no and ‘?’ indicates unclear.

### Primary outcome

All studies reported worse absolute postoperative scores among patients prescribed preoperative opioids compared to opioid-naïve patients (Table 3). Of the studies that reported a parameter of statistical significance comparing absolute postoperative PRO scores between the two groups, all reported worse scores among opioid users when compared to opioid-naïve patients (range 4.7–13 points, *p* < 0.05 for all) (Additional file 2: Table S2). When relative change in PRO score was analyzed, five of the six studies demonstrated that opioid users had a smaller change in PRO scores when compared to opioid-naïve patients (range 2.4–20.2 points). Of the three studies that performed statistical analysis comparing the change in PRO score between groups, all reported these differences to be statistically significant (p < 0.05 for all) (Additional file 2: Table S2).

**Table 3:** Comparison of scores between patient prescribed preoperative opioids and opioid-naïve patients

Study	Patients	PRO	Statistic	Preoperative Score		Postoperative Score		Mean Change^a^		Difference^b^
				OU	nOU	OU	nOU	OU	nOU	(OU – nOU)
Zywiel et al.	OU (n) = 45 nOU (n) = 45	KSS	mean (SD)	38.0	37.0	79.0 (10.0)	92.0 (10.0)	41.0 (14.5)	55.0 (12.0)	14.0
Smith et al.	OU (n) = 36 nOU (n) = 120	WOMAC Pain	mean (SD)	55.4	56.3	82.9 (12.7)	89.5 (12.7)	27.0 (12.7)	33.6 (12.7)	6.6
Franklin et al.	OU (n) = 1544 nOU (n) = 4802	KSS	mean (SD)	34.8	37.1	81.3 (15.7)	86.0 (14.1)	46.5 (15.4)	48.9 (14.9)	2.4
Pivec et al.	OU (n) = 54 nOU (n) = 54	HHS	mean (SD)	43.0	45.0	84.0 (11.5)	91.0 (11.5)	41.0 (81.2)	46.0 (91.1)	5.0
Nguyen et al.	OU (n) = 41 nOU (n) = 41	WOMAC	mean (SD)	47.5	44.1	65.3 (35.1)	83.1 (35.1)	17.8 (41.8)	39.0 (41.8)	20.2
Goesling et al.	OU (n) = 111 nOU (n) = 313	WOMAC	mean (SD)	39.3	49.4.0	80.8 (17.3)	85.5 (12.8)	41.5 (16.2)	36.1 (13.8)	- 5.4

Abbreviations

*PRO* Joint or Disease Specific Patient-Reported Outcome Score. All scores Transformed to a 0 to 100-point scale (100 indicating the best possible score), *WOMAC* The Western Ontario and McMaster Universities Osteoarthritis Index, *KSS* Knee Society Score, *HHS* Harris Hip Score, *OU* Patients prescribed preoperative opioids, *nOU* Preoperative Opioid-naïve patients, *n* Number of patients, *SD* Standard deviations, *CI* Confidence Interval

Notes

^a^Mean change calculated by the difference in preoperative and postoperative score.

^b^Difference represents the mean difference between opioid users and non-opioid users with a positive indicating benefit for preoperative opioid-naïve patients.

**Fig. 2:**
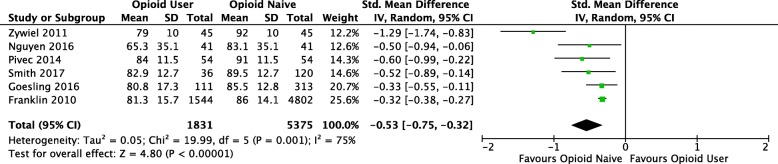
Forest plot comparing absolute PRO scores between opioid users and opioid-naïve-patients (CI, confidence interval; IV, Inverse variance; Random, random effects model; SMD, standard mean difference; SD, standard deviation. Individual studies SMD; pooled SMD)

**Fig. 3:**
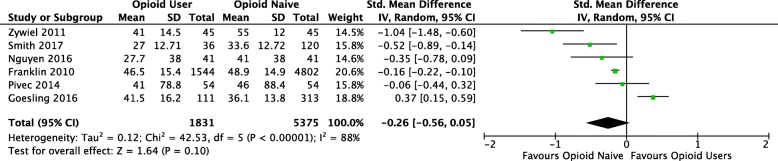
Forrest plot comparing change in PRO scores between opioid users and opioid-naïve patients. Change in PRO score calculated by the difference in preoperative PRO score and postoperative PRO scores (CI, confidence interval; IV, Inverse variance; Random, random effects model; SMD, standard mean difference; SD, standard deviation. Individual studies SMD; pooled SMD)

Our meta-analysis found that opioid users had worse absolute postoperative PRO scores, compared to opioid-naïve patients (SMD -0.53, 95% CI -0.75, − 0.32, *p* < 0.0001) (Fig. 2). Based on Cohen’s coefficient, the effect size is moderate. Contrary to individual study results, relative change in PRO did not reach statistical significance between groups (SMD -0.26, 95% CI -0.55, 0.05, *p* = 0.10) (Fig. 3) in the meta-analysis; the effect size was also considered small. However, heterogeneity was statistically high between studies for both change in PRO score (I^2^_change_ = 88%), and absolute postoperative PRO score (I^2^_absolute_ = 75%). Subgroup analysis did not influence the magnitude or significance of the results when stratified by joint (knee or hip) or by WOMAC domain score (data not shown).

### Secondary outcomes

#### Opioid use prior to TJA

The prevalence of opioid use prior to TJA was 24.4% when data from studies were pooled (range 24 to 29%) (Table 4). Only two studies reported a mean dose for opioid users; Zywiel et al. (2011) reported the mean preoperative dose was 58 mg morphine equivalents per day (MED) (range 20–300 mg MED), while Nguyen et al. (2011) reported 34% of patients’ preoperative dose was < 30 mg MED, 17% 31–60 mg MED, 15% 61–120 mg MED, and 34% had > 120 mg MED [27, 31].

**Table 4:** Preoperative opioid use definitional parameters

Study	Definition of Opioid User	Justification	Opioid Use	Source of Pharmacy Data	Included Opioids	Preoperative Duration	Preoperative Dose (MED)
Zywiel et al.	Any documented opioid use (minimum ≥20 mg morphine equivalents per day) for minimum 6 weeks prior to index procedure	Chu et al (2006)	N/A	Prescription records, clinic notes and admission records	N/A	Minimum 6 weeks	58 mg
Smith et al.	At least 1 opioid prescription within 2 years of index surgery	N/A	23%	Clinical visit notes, anesthesiology reports, discharge notes, prescription history, and medication lists.	Oxycodone, hydrocodone, hydromorphone, morphine, tramadol, codeine	N/A	N/A
Franklin et al.	Any documented opioid prescription prior to index procedure	N/A	24%	Administrative Database	Percocet, Vicodin, Darvocet, Tylenol with codeine ‘other’	N/A	N/A
Pivec et al.	Minimum of 6 weeks of narcotic use (minimum ≥30 mg morphine equivalents per day) prior to index TKA	Chu et al (2006)	N/A	Clinic charts, in-patient hospital medication administration records, prescription documentation, and phone interviews	Morphine, codeine, hydrocodone, hydromorphone, methadone, meperidine, oxycodone, propoxyphene, tramadol, transdermal fentanyl	Minimum 6 weeks	N/A
Nguyen et al.	Continuous opioid use for at least 4 weeks prior to index procedure	Chu et al (2006)	N/A	Clinic and referral notes	N/A	Minimum 4 weeks	^a^ Low 34% Medium 17% High 15% Very High 34%
Goesling et al.	Patient-reported opioids use prior to index procedure	N/A	29%	Chart review, confirmed by patient	N/A	N/A	N/A
		Mean ^b^	24%				

Abbreviations

*N/A*, data not available, *MED* Morphine equivalent dose

Notes

^a^Classification of opioid user: Low (< 30 mg), Medium (31–60 mg) High (61 - 120 mg) and Very High (> 121 mg).

^b^Mean calculated by summing number of patients prescribed preoperative opioids (*n* = 1747) and dividing by total patients (*n* = 7163).

Chu et al. (2006) – minimum duration and dosage required of morphine required to develop opioid induced hyperalgesia

#### Preoperative opioid use definitional parameters

Definitional parameters for preoperative use ranged from “any” documented opioid use within two years of the index surgery to “a minimum of six weeks” of opioid use prior to index procedure (Table 4). Three studies justified their case definition based on the minimum amount of time required to develop opioid induced hyperalgesia (OIH); the three remaining studies did not include a justification (Table 4). Two studies reported that the minimum preoperative dose for the patients to be classified as opioid users was 20 or 30 mg MED, respectively (Table 4). Of the three studies that outlined which opioids were included in their study, only two considered Tramadol as an opioid (Table 4).

#### Postoperative opioid use

Pivec et al. (2014*)* reported opioid users consumed significantly more opioids on postoperative days 0, 1 and 3, and at six weeks compared to opioid-naïve patients (*p* < 0.05 for all) [29]. But, Zywiel et al. (2011) reported that there were no significant differences in mean MED at discharge from TKA when comparing opioid users to opioid-naïve patients (85 mg vs 91 mg MED, *p* = 0.95). Opioid users were also found to have higher rates of persistent postoperative opioid use at long-term follow up after TJA compared to opioid-naïve patients (Additional file 2: Table S3). At six months’ follow up, Goesling et al. (2016) noted 50.3% of TKA and 37.7% of THA preoperative opioid users were still prescribed opioids, compared to only 8.2% of TKA and 4.3% of THA opioid-naïve patients (*p* < 0.01 for both). At 12-months follow up, Franklin et al. (2010) reported that 14% of opioid users were still using opioids compared to 2.6% of opioid-naïve patients (*p* < 0.01). At final follow up (mean 58 months), Pivec et al. (2014) reported that 19% of opioid users were still prescribed opioids, compared to 4% of opioid-naïve patients (*p* = 0.04).

#### Impact of patient characteristics

There were significant differences in preoperative patient characteristics between opioid users and opioid-naïve patients (Table 5). Of the three studies that did not match for age, two reported that opioid users were younger than opioid-naïve patients (p < 0.01 for both) (Table 5). All studies reported that opioid users had worse preoperative mental health when compared to opioid-naïve patients. Goesling et al. (2016) reported that opioid users had worse hospital anxiety and depression scale (HADS) depression scores, HADS anxiety scores and catastrophizing scores when compared to opioid-naïve patients (p < 0.01 for all). Likewise, Smith et al. (2017) reported that opioid users had worse pain catastrophizing scores and Franklin et al. (2010) found opioid users had worse SF-12 mental component scores preoperatively when compared to opioid-naïve patients (*p* < 0.05 for both). Finally, Zywiel et al. (2011) found significantly more opioid users prescribed antidepressants or anxiolytics preoperatively, compared to opioid-naïve patients (21 patients vs. 10 patients, *p* = 0.014) and Pivec et al. (2014) reported opioid users also had significantly higher numbers of a past psychiatric diagnosis than opioid-naïve patients (16 patients vs. 7 patients, *p* = 0.03). Despite these group differences, there was no difference in the number of patients with chronic back pain, actively smoking or reporting alcohol use when groups were compared in both studies (*p* > 0.05 for all).

**Table 5:** Comparison of preoperative patient demographic between patient prescribed preoperative opioids and opioid-naïve patients

Study	Patient Characteristics	OU	nOU	p
Zywiel et al.	Mean age^a^	56	57	0.653
	% Male^a^	31.1	31.1	–
	Mean BMI^a^	34	34	0.884
	Number of patients prescribed antidepressants or anxiolytics	21	10	0.014
	Number of patients with chronic back pain or prior back surgery	9	8	0.788
	Number of patients actively smoking	10	7	0.419
	Number of patients reporting alcohol use	0	1	0.316
	Number of patients with systemic corticosteroid use	8	7	0.777
Smith et al.	Mean age	67.5	65.2	0.13
	% Female	23.7	76.3	0.81
	Mean BMI	31.0	31.1	0.84
	Mean comorbidities	0.81	0.81	0.91
	Preoperative Pain Catastrophizing Scale (SD)	15.3 (10.3)	10.7 (7.7)	0.006
	Mean unadjusted preoperative WOMAC Pain (SD)	53.1 (15.7)	57 (12.8)	0.12
	Mean unadjusted preoperative WOMAC Function (SD)	51.0 (14.1)	57.9 (13.8)	0.009
Franklin et al.	Mean age (SD)	65.3 (11.0)	68.1 (9.7)	< 0.001
	% Male	28.9	34.1	< 0.001
	Mean BMI	32.6 (7.5)	31.7 (6.8)	< 0.001
	Mean SF-12 PCS (SD)	28.2 (7.1)	30.6 (7.9)	< 0.001
	Mean SF-12 MCS (SD)	48.7 (12.0)	53.0 (10.8)	< 0.001
Pivec et al.	Mean age^a^	55	55	–
	% Male^a^	54	54	–
	BMI^a^	30.2	29.9	–
	Number of patients with history of a psychiatric diagnosis	16	7	0.03
	Number of patients with history of alcohol abuse	7	6	0.77
	Number of patients reporting active smoking	14	12	0.83
	Number of patients with history of back pain	11	14	0.24
	Number of patients with history of back surgery	7	10	0.60
	Number of patients with systemic corticosteroid use	10	6	0.42
	Numbers of patients reporting worker’s compensation	2	1	0.56
Nguyen et al.	Mean age^a^	60	58	–
	% Male^a^	34	34	–
	Mean SF-12 MCS	42.8	49.1	–
	Mean SF-12 PCS	28.8	30.9	–
Goesling et al.	Mean age	59.3	63.6	< 0.001
	% Male	43.1	50.1	0.127
	BPI Overall Pain Severity (SD)	5.6 (1.8)	4.3 (12.0)	< 0.001
	HADS Depression (SD)	5.9 (3.5)	4.2 (3.2)	< 0.001
	HADS Anxiety (SD)	6.2 (3.8)	5.2 (3.6)	0.002
	CSQ Catastrophizing (SD)	6.5 (5.8)	4.2 (5.7)	0.001

Abbreviations

*SD* Standard deviation, *WOMAC* The Western Ontario and McMaster Universities Osteoarthritis Index, *KSS* Knee Society Score, *HHS* Harris Hip Score, *OU* Patients prescribed preoperative opioids, *nOU* Preoperative opioid-naïve patients, *BPI* Brief Pain Inventory, *HADS* Hospital Anxiety and Depression Scale Depression, *CSQ* Coping Strategies Questionnaire, ‘-‘not reported in study

Notes

^a^Matched Cohort

#### Length of stay and discharge characteristics

Two studies reported varying effects on postoperative health services (Additional file 2: Table S3). While both studies found the mean hospital length of stay increased when opioid users were compared to opioid-naïve patients, only one study reported a statistically significant result (Additional file 2: Table S3). Although preoperative opioid use did not affect discharge destination from the surgical hospital, opioid users were more likely to be referred to chronic pain clinic postoperative when compared to preoperative opioid-naïve patients (8 patients vs. 1 patient, *p* < 0.001) [31].

## Discussion

In our pooled analysis comparing preoperative opioid users to opioid-naïve patients, we found that opioid users had worse absolute postoperative PRO scores, but similar relative change in PRO scores when compared to opioid-naïve patients (Figs. 2 and 3). These results suggest that patients prescribed opioids preoperatively experience the same level of improvement compared to their opioid-naïve counterparts but still have overall worse PRO scores. Morris et al. (2016) also reported that patients prescribed opioids prior to total shoulder arthroplasty achieved similar relative change in PRO scores postoperatively, but worse overall benefit when compared to opioid-naïve patients [14, 32]. These two studies also reported that significantly fewer patients prescribed preoperative opioids were satisfied with their surgery postoperatively, compared to opioid-naïve patients (80% vs 91%, *p* = 0.03) [32]. It has been hypothesized that OIH may explain the differences between these two groups [27, 29, 31, 33]. OIH is a process by which patients taking long-term opioids have a paradoxical increased response to painful stimuli [33]. However, the reasons why these changes persist at long-term follow up (> 6 months) is uncertain and likely relates to the complex relationship between chronic pain, opioid use and patient’s psychological factors [34].

Patients with mental health conditions, such as depression and anxiety are more likely to be prescribed opioids, at higher doses and for longer durations [35, 36]. Our results were consistent with these reports; more opioid users reported psychiatric conditions, antidepressant or anxiolytic use than those who were opioid-naïve (Table 5). Understanding the association between opioids use and depression is complex, as they often coexist and can be a cause, or result of the other [35, 37, 38]. Not only have studies reported prolonged opioid use can induce depression, but depressed patients more frequently seek medical attention for pain, and are three times more likely to be prescribed chronic opioid therapy (> 90 days) [34, 35, 38]. Despite this association, Smith et al. (2017) reported that after adjusting for these group differences, preoperative opioid was still associated with worse postoperative PRO scores after TKA [10].

The search strategy was not designed to exhaustively review our secondary outcomes, but our results did highlight several important points regarding opioid prescribing practices among TJA patients. First, a substantial number of patients (24%) are prescribed opioids prior to TJA in the USA (Table 4). To our knowledge, only two studies have reported the prevalence of preoperative opioid use outside of the USA; 5% of patients awaiting TKA, and 6% of patients awaiting THA were considered opioid users prior to surgery in Australia [39, 40]. Our critical analysis describing the parameters used to define opioid users demonstrated definitional differences are likely contributing to the variation in preoperative opioid prescription rates (Table 4). In addition, there was an inconsistent inclusion of Tramadol, one of the most commonly prescribed opioids (Table 4). This exclusion may be explained by previous American Academy of Orthopaedic Surgeons guidelines that recommended its use for the management of pain associated with knee osteoarthritis [8, 41]. However, Tramadol is now routinely classified as an opioid in national prescribing guidelines as the drug shares similar abuse rates and side effects as traditional opioids [6, 42, 43]. Collectively, the observed variations in case definitions create uncertainty about the true prevalence of preoperative opioid rates among patients undergoing TJA.

We also noted that patients prescribed preoperative opioids are more likely to continue to use opioids at long-term follow up after surgery when compared to preoperative opioid-naïve patients (Additional file 2: Table S3). These results are consistent with a study that reported preoperative opioid use (> 225 days), depression and pain catastrophizing was associated with persistent postoperative opioid use after THA [28, 39]. These patient factors may explain the subset of preoperative opioid-naïve patients that go on to long-term opioid use postoperatively, and underscores the importance of opioid stewardship. Implementing standardized, evidence-based postoperative opioid prescribing protocols may optimize postoperative opioid prescriptions and are particularly important for patients at risk for transitioning from short-term to long-term opioid therapy postoperatively [39, 44, 45].

The main limitation of this systematic review was the low number of studies available that used different analytic approaches, outcomes measures and follow-up periods. Given these differences, we used a random effects model that accounts for statistical heterogeneity between the studies and provides a more conservative estimate of the significance than a fixed effects model [18]. In addition, sensitivity analysis for the estimations, including score construct (pain or total score), surgical joint (hip or knee) were robust and did not significantly change the results.

## Conclusion

To our knowledge, this is the first systematic review comparing the impact of preoperative opioid use on PRO after TJA. Our study demonstrated that patients prescribed preoperative opioids may attain worse overall pain and function benefits after TJA, compared to opioid-naïve patients, but do still benefit from undergoing TJA. However, without further research that considers other patient factors in the context of preoperative opioid use, our understanding of the independent impact of opioid use on outcomes after surgery remains uncertain.

## Additional files

Additional file 1: Appendix A: Database search strategies. Appendix B: JBI Critical Appraisal Checklist for Cohort Studies. (DOCX 151 kb)

Additional file 2: **Table S1.** Additional Data provided for Goesling *et at* (2016). **Table S2.** Original Extracted Patient-Reported Outcome Scores. **Table S3.** Comparison of Secondary Outcomes between Patient Prescribed Preoperative Opioids and Opioid-Naïve Patients. (DOCX 29 kb)

## Abbreviations

PRO: Patient reported outcomes
TJA: Total Joint Arthroplasty
