# Supplementary material for: Preoperative opioid use is associated with worse patient outcomes after Total joint arthroplasty: a systematic review and meta-analysis
Source: BMC Musculoskelet Disord. 2019 May 18;20:234. doi: 10.1186/s12891-019-2619-8 (PMC6525974; doi:10.1186/s12891-019-2619-8)
Supplement: Supplementary file 1 — Appendix A: Database search strategies. Appendix B: JBI Critical Appraisal Checklist for Cohort Studies. (DOCX 151 kb) [file 12891_2019_2619_MOESM1_ESM.docx]

Additional file 1

**APPENDIX A:  Database search strategies**

**Ovid MEDLINE(R) Epub Ahead of Print, In-Process & Other Non-Indexed Citations, Ovid MEDLINE(R) Daily and Ovid MEDLINE(R) <1946 to Present>**

--------------------------------------------------------------------------------

1     exp Arthroplasty, Replacement/ or exp Arthroplasty/ or (arthroplasty or ((joint or knee* or hip* or shoulder*) adj2 replacement)).mp.

2     exp narcotics/ or exp analgesics, opioid/

3     (opiate* or opioid* or narcotic* or morphin* or duramorph or ms contin or morphia or oramorph sr or sdz 202 250 or sdz202250 or sdz202 250 or alfenta or alfentanil or fanaxal or limifen or rapifen or r 39209 or r39209 or alphaprodine or Nisentil or prodine or buprenorphine or buprenex or buprex or prefin or subutex or temgesic or 6029 m or 6029m or rx6029m).mp.

4     (butorphanol or dolorex or moradol or stadol or torbugesic or bc 2627 or bc2627 or codeine or ardinex or idocodeine or n methylmorphine or Dextromoramide or d moramide or palfium or pyrrolamidol or Dextropropoxyphene or d propoxyphene or darvon or propoxyphene or Enkephalin or dago or dagol or damge or damgo or rx 783006 or dpdpe).mp.

5     (fentanyl or duragesic or durogesic or fentanest or fentora or phentanyl or r 4263 or r4263 or sublimaze or hydrocodon* or codinovo or dicodid or dihydrocodeinone or hycodan or hycon or hydrocodeinonebitartrate or robidone or hydromorphon* or dihydromorphinone or dilaudid or laudacon or palladone).mp.

6     (meperidine or demerol or dolantin or dolargan or dolcontral or dolin or dolosal or dolsin or isonipecain or lidol or lydol or operidine or pethidine or Meptazinol or meptid or wy 22811 or wy22811 or nalbuphine or nubain or en 2234a or en2234a or oxycodone or dihydrohydroxycodeinone or dihydrone or dinarkon or eucodal or oxiconum or oxycodeinon or oxycone or oxycontin or pancodine or theocodin or percocet).mp.

7     (oxymorphone or numorphan or opana or pentazocine or fortral or lexir or talwin or phenoperidine or fenoperidine or lealgin or operidine or r 1406 or r1406 or pirinitramid* or piritramid* or dipidolor or dipydolor or promedol or dimethylmeperidine or isopromedol or trimeperidine or sufentanil or sufentanilhameln or sulfentanil or sulfentanyl or r 30730 or r30730).mp.

8     (tramadol or adolonta or amadol or biodalgic or biokanol or contramal or jutadol or k 315 or k315 or mtwtramadol or nobligan or prontofort or ranitidin 1a pharma or takadol or theradol or tiral or topalgic or Tradol or tradolpuren or tradonal or tralgiol or trama or tramadorsch or tramabeta or tramadin or tramadoc or tramadoldolgit or tramadolhameln or tramadolor or tramadolratiopharm or tramadura or tramagetic or tramagit or tramake or tramal or tramex or tramundin or trasedal or ultram or xymel 50 or zamudol or zumalgic or zydol or zytram).mp.

9     (acetorophine or acetylcodeine or acetymethadol or anileridine or apadoline or azidomorphine or benzhydrocodone or bezitramide or bremazocine or brompton mixture or ciramadol or cocomadol or codydramol or conorfone or cyclazocine or dextrorphan or dezocine or diamorphine or diconal or dihydroetorphine or dihydromorphine or dimethylthiambutene or dipipanone or dynorphin or enadoline or eptazocine or ethylketazocine or ethylmorphine or etonitazene or etorphine or etoxeridine or faxeladol or furethidine or gelonida or isalmadol or isomethodone or ketazocine or ketobemidone or ketogan or kyotorphin or lefetamine or levacetylmethadol or levomethadone or levorphanol or metazocine or methylsamidorphan or tilidine or nicodine or nicomorphine or noracymethadol or bufigen or nubain* or nalbufin* or nalcryn or nalpain or onfor or noracymethadol or norbuprenorphine or normorphine or norpethidine or norpropoxyphene or nortramadol or oliceridine or oripavine or pentamorphone or phenadoxone or phencyclidine or picenadol or piminodine or piritramide or profadol or propiram or sameridine or samidorphan or semorphone or tapentadol or thebaine or tifluadom or tilidine or tonazocine or vicodin).mp.

10     or/2-9

11     1 and 10

12     exp preoperative care/ or preoperative period/

13     (preoperativ* or pre-operativ* or pre-surg* or presurg*).mp.  or ((prior or "before") adj3 (surg* or arthroplasty or replacement)).ti,ab,kf.

14     12 or 13

15     11 and 14

**Embase <1974 to Current>**

--------------------------------------------------------------------------------

1     exp arthroplasty/ or (arthroplasty or ((joint or knee* or hip* or shoulder*) adj2 replacement)).ti,ab,kw.

2     exp narcotic agent/ or exp narcotic analgesic agent/

3     (opiate* or opioid* or narcotic* or morphin* or duramorph or ms contin or morphia or oramorph sr or sdz 202 250 or sdz202250 or sdz202 250 or alfenta or alfentanil or fanaxal or limifen or rapifen or r 39209 or r39209 or alphaprodine or Nisentil or prodine or buprenorphine or buprenex or buprex or prefin or subutex or temgesic or 6029 m or 6029m or rx6029m).ti,ab,kw.

4     (butorphanol or dolorex or moradol or stadol or torbugesic or bc 2627 or bc2627 or codeine or ardinex or idocodeine or n methylmorphine or Dextromoramide or d moramide or palfium or pyrrolamidol or Dextropropoxyphene or d propoxyphene or darvon or propoxyphene or Enkephalin or dago or dagol or damge or damgo or rx 783006 or dpdpe).ti,ab,kw.

5     (fentanyl or duragesic or durogesic or fentanest or fentora or phentanyl or r 4263 or r4263 or sublimaze or hydrocodon* or codinovo or dicodid or dihydrocodeinone or hycodan or hycon or hydrocodeinonebitartrate or robidone or hydromorphon* or dihydromorphinone or dilaudid or laudacon or palladone).ti,ab,kw.

6     (meperidine or demerol or dolantin or dolargan or dolcontral or dolin or dolosal or dolsin or isonipecain or lidol or lydol or operidine or pethidine or Meptazinol or meptid or wy 22811 or wy22811 or nalbuphine or nubain or en 2234a or en2234a or oxycodone or dihydrohydroxycodeinone or dihydrone or dinarkon or eucodal or oxiconum or oxycodeinon or oxycone or oxycontin or pancodine or theocodin or percocet).ti,ab,kw.

7     (oxymorphone or numorphan or opana or pentazocine or fortral or lexir or talwin or phenoperidine or fenoperidine or lealgin or operidine or r 1406 or r1406 or pirinitramid* or piritramid* or dipidolor or dipydolor or promedol or dimethylmeperidine or isopromedol or trimeperidine or sufentanil or sufentanilhameln or sulfentanil or sulfentanyl or r 30730 or r30730).ti,ab,kw.

8     (tramadol or adolonta or amadol or biodalgic or biokanol or contramal or jutadol or k 315 or k315 or mtwtramadol or nobligan or prontofort or ranitidin 1a pharma or takadol or theradol or tiral or topalgic or Tradol or tradolpuren or tradonal or tralgiol or trama or tramadorsch or tramabeta or tramadin or tramadoc or tramadoldolgit or tramadolhameln or tramadolor or tramadolratiopharm or tramadura or tramagetic or tramagit or tramake or tramal or tramex or tramundin or trasedal or ultram or xymel 50 or zamudol or zumalgic or zydol or zytram).ti,ab,kw.

9     (acetorophine or acetylcodeine or acetymethadol or anileridine or apadoline or azidomorphine or benzhydrocodone or bezitramide or bremazocine or brompton mixture or ciramadol or cocomadol or codydramol or conorfone or cyclazocine or dextrorphan or dezocine or diamorphine or diconal or dihydroetorphine or dihydromorphine or dimethylthiambutene or dipipanone or dynorphin or enadoline or eptazocine or ethylketazocine or ethylmorphine or etonitazene or etorphine or etoxeridine or faxeladol or furethidine or gelonida or isalmadol or  isomethodone or ketazocine or ketobemidone or ketogan or kyotorphin or lefetamine or levacetylmethadol or levomethadone or levorphanol or metazocine or methylsamidorphan or tilidine or nicodine or nicomorphine or noracymethadol or bufigen or nubain* or nalbufin* or nalcryn or nalpain or onfor or noracymethadol or norbuprenorphine or normorphine or norpethidine or norpropoxyphene or nortramadol or oliceridine or oripavine or pentamorphone or phenadoxone or phencyclidine or picenadol or piminodine or piritramide or profadol or propiram or sameridine or samidorphan or semorphone or tapentadol or thebaine or tifluadom or tilidine or tonazocine or vicodin).ti,ab,kw.

10     or/2-9

11     1 and 10

12     exp preoperative chemotherapy/ or exp preoperative care/ or exp preoperative period/ or exp preoperative treatment/

13     (preoperativ* or pre-operativ* or pre-surg* or presurg*).ti,ab,kw. or ((prior or  "before") adj3 (surg* or arthroplasty or replacement)).ti,ab,kw.

14     12 or 13

15     11 and 14

**Cochrane Library**

#1     [mh "Arthroplasty, Replacement"] or [mh "Arthroplasty"] or (arthroplasty or ((joint or knee* or hip* or shoulder*) near/2 replacement)):ti,ab,kw

#2       [mh "narcotics"] or [mh "analgesics,opioid"]

#3  (opiate* or opioid* or narcotic* or morphin* or duramorph or "ms contin" or morphia or oramorph or "sdz 202 250" or sdz202250 or "sdz202 250" or alfenta or alfentanil or fanaxal or
limifen or rapifen or "r 39209" or r39209 or alphaprodine or nisentil or prodine or buprenorphine or buprenex or buprex or prefin or subutex or temgesic or "6029 m" or 6029m or rx6029m):ti,ab,kw

#4  (butorphanol or dolorex or moradol or stadol or torbugesic or "bc 2627" or bc2627 or codeine or ardinex or idocodeine or methylmorphine or Dextromoramide or moramide or palfium or pyrrolamidol or dextropropoxyphene or propoxyphene or darvon or propoxyphene or enkephalin or dago or dagol or damge or damgo or "rx 783006" or rx783006 or dpdpe):ti,ab,kw

#5 (fentanyl or duragesic or durogesic or fentanest or fentora or phentanyl or "r 4263" or r4263 or sublimaze or hydrocodon* or codinovo or dicodid or dihydrocodeinone or hycodan or hycon or hydrocodeinonebitartrate or robidone or hydromorphon* or dihydromorphinone or
dilaudid or laudacon or palladone):ti,ab,kw

#6 (meperidine or demerol or dolantin or dolargan or dolcontral or dolin or dolosal or dolsin or isonipecain or lidol or lydol or operidine or pethidine or meptazinol or meptid or "wy 22811" or
wy22811 or nalbuphine or nubain or "en 2234a" or en2234a or oxycodone or dihydrohydroxycodeinone or dihydrone or dinarkon or eucodal or oxiconum or oxycodeinon or oxycone or oxycontin or pancodine or theocodin or percocet):ti,ab,kw
#7 (oxymorphone or numorphan or opana or pentazocine or fortral or lexir or talwin or phenoperidine or fenoperidine or lealgin or operidine or "r 1406" or r1406 or pirinitramid* or piritramid* or dipidolor or dipydolor or promedol or dimethylmeperidine or isopromedol or
trimeperidine or sufentanil or sufentanilhameln or sulfentanil or sulfentanyl or "r 30730" or r30730):ti,ab,kw

#8 (tramadol or adolonta or amadol or biodalgic or biokanol or contramal or jutadol or "k 315" or k315 or mtwtramadol or nobligan or prontofort or ranitidin or takadol or theradol or tiral or topalgic or tradol or tradolpuren or tradonal or tralgiol or trama or tramadorsch or tramabeta or tramadin or tramadoc or tramadoldolgit or tramadolhameln or tramadolor or tramadolratiopharm or tramadura or tramagetic or tramagit or tramake or tramal or tramex or tramundin or trasedal or ultram or xymel or zamudol or zumalgic or zydol or zytram):ti,ab,kw

#9  (acetorophine or acetylcodeine or acetymethadol or anileridine or apadoline or azidomorphine or benzhydrocodone or bezitramide or bremazocine or "brompton mixture" or ciramadol or cocomadol or codydramol or conorfone or cyclazocine or dextrorphan or dezocine or diamorphine or diconal or dihydroetorphine or dihydromorphine or dimethylthiambutene or dipipanone or dynorphin or enadoline or eptazocine or ethylketazocine or ethylmorphine or etonitazene or etorphine or etoxeridine or faxeladol or furethidine or gelonida or isalmadol or

isomethodone or ketazocine or ketobemidone or ketogan or kyotorphin or lefetamine or levacetylmethadol or levomethadone or levorphanol or metazocine or methylsamidorphan or tilidine or nicodine or nicomorphine or noracymethadol or bufigen or nubain* or nalbufin* or nalcryn or nalpain or onfor or noracymethadol or norbuprenorphine or normorphine or norpethidine or norpropoxyphene or nortramadol or oliceridine or oripavine or pentamorphone or phenadoxone or phencyclidine or picenadol or piminodine or piritramide or profadol or propiram or sameridine or samidorphan or semorphone or tapentadol or thebaine or tifluadom or tilidine or tonazocine or vicodin):ti,ab,kw

#10 #2 or #3 or #4 or #5 or #6 or #7 or #8 or #9

#11    #1 and #10

#12 [mh "preoperative care"] or [mh "preoperative period"] or (preoperativ* or "pre-operativ*" or "pre-surg*" or presurg*):ti,ab,kw OR ((prior or  "before") NEAR/3 (surg* or arthroplasty or replacement)):ti,ab,kw

#13 #11 and #12

**Scopus**

TITLE-ABS-KEY(arthroplasty or ((joint or knee* or hip* or shoulder*) w/2 replacement)) and TITLE-ABS-KEY(opiate* or opioid* or narcotic* or morphin* or duramorph or "ms contin" or morphia or oramorph or "sdz 202 250" or sdz202250 or "sdz202 250" or alfenta or alfentanil or fanaxal or limifen or rapifen or "r 39209" or r39209 or alphaprodine or nisentil or prodine or buprenorphine or buprenex or buprex or prefin or subutex or temgesic or "6029 m" or 6029m or rx6029m or butorphanol or dolorex or moradol or stadol or torbugesic or "bc 2627" or bc2627 or codeine or ardinex or idocodeine or methylmorphine or dextromoramide or moramide or palfium or pyrrolamidol or dextropropoxyphene or propoxyphene or darvon or propoxyphene or enkephalin or dago or dagol or damge or damgo or "rx 783006" or rx783006 or dpdpe or fentanyl or duragesic or durogesic or fentanest or fentora or phentanyl or "r 4263" or r4263 or sublimaze or hydrocodon* or codinovo or dicodid or dihydrocodeinone or hycodan or hycon or hydrocodeinonebitartrate or robidone or hydromorphon* or dihydromorphinone or dilaudid or laudacon or palladone or meperidine or demerol or dolantin or dolargan or dolcontral or dolin or dolosal or dolsin or isonipecain or lidol or lydol or operidine or pethidine or meptazinol or meptid or "wy 22811" or wy22811 or nalbuphine or nubain or "en 2234a" or en2234a or oxycodone or dihydrohydroxycodeinone or dihydrone or dinarkon or eucodal or oxiconum or oxycodeinon or oxycone or oxycontin or pancodine or theocodin or percocet or oxymorphone or numorphan or opana or pentazocine or fortral or lexir or talwin or phenoperidine or fenoperidine or lealgin or operidine or "r 1406" or r1406 or pirinitramid* or piritramid* or dipidolor or dipydolor or promedol or dimethylmeperidine or isopromedol or trimeperidine or sufentanil or sufentanilhameln or sulfentanil or sulfentanyl or "r 30730" or r30730 or tramadol or adolonta or amadol or biodalgic or biokanol or contramal or jutadol or "k 315" or k315 or mtwtramadol or nobligan or prontofort or ranitidin or takadol or theradol or tiral or topalgic or tradol or tradolpuren or tradonal or tralgiol or trama or tramadorsch or tramabeta or tramadin or tramadoc or tramadoldolgit or tramadolhameln or tramadolor or tramadolratiopharm or tramadura or tramagetic or tramagit or tramake or tramal or tramex or tramundin or trasedal or ultram or xymel or zamudol or zumalgic or zydol or zytram or acetorophine or acetylcodeine or acetymethadol or anileridine or apadoline or azidomorphine or benzhydrocodone or bezitramide or bremazocine or "brompton mixture" or ciramadol or cocomadol or codydramol or conorfone or cyclazocine or dextrorphan or dezocine or diamorphine or diconal or dihydroetorphine or dihydromorphine or dimethylthiambutene or dipipanone or dynorphin or enadoline or eptazocine or ethylketazocine or ethylmorphine or etonitazene or etorphine or etoxeridine or faxeladol or furethidine or gelonida or isalmadol or

isomethodone or ketazocine or ketobemidone or ketogan or kyotorphin or lefetamine or levacetylmethadol or levomethadone or levorphanol or metazocine or methylsamidorphan

or tilidine or nicodine or nicomorphine or noracymethadol or bufigen or nubain* or nalbufin* or nalcryn or nalpain or onfor or noracymethadol or norbuprenorphine or normorphine or norpethidine or norpropoxyphene or nortramadol or oliceridine or oripavine or pentamorphone or phenadoxone or phencyclidine or picenadol or piminodine or piritramide or profadol or propiram or sameridine or samidorphan or semorphone or tapentadol or thebaine or tifluadom or tilidine or tonazocine or vicodin) and TITLE-ABS-KEY(preoperativ* or "pre-operativ*" or "pre-surg*" or presurg* or ((prior or  "before") pre/3 (surg* or arthroplasty or replacement)))

**Web of Science Core Collection**

#1 TS=(arthroplasty or (joint or knee* or hip* or shoulder*) near/2 replacement)

#2  TS=(opiate* or opioid* or narcotic* or morphin* or duramorph or "ms contin" or morphia or oramorph or "sdz 202 250" or sdz202250 or "sdz202 250" or alfenta or alfentanil or fanaxal or limifen or rapifen or "r 39209" or r39209 or alphaprodine or nisentil or prodine or buprenorphine or buprenex or buprex or prefin or subutex or temgesic or "6029 m" or 6029m or rx6029m or butorphanol or dolorex or moradol or stadol or torbugesic or "bc 2627" or bc2627 or codeine or ardinex or idocodeine or methylmorphine or dextromoramide or moramide or palfium or pyrrolamidol or dextropropoxyphene or propoxyphene or darvon or propoxyphene or enkephalin or dago or dagol or damge or damgo or "rx 783006" or rx783006 or dpdpe or fentanyl or duragesic or durogesic or fentanest or fentora or phentanyl or "r 4263" or r4263 or sublimaze or hydrocodon* or codinovo or dicodid or dihydrocodeinone or hycodan or hycon or hydrocodeinonebitartrate or robidone or hydromorphon* or dihydromorphinone or dilaudid or laudacon or palladone or meperidine or demerol or dolantin or dolargan or dolcontral or dolin or dolosal or dolsin or isonipecain or lidol or lydol or operidine or pethidine or meptazinol or meptid or "wy 22811" or wy22811 or nalbuphine or nubain or "en 2234a" or en2234a or oxycodone or dihydrohydroxycodeinone or dihydrone or dinarkon or eucodal or oxiconum or oxycodeinon or oxycone or oxycontin or pancodine or theocodin or percocet or oxymorphone or numorphan or opana or pentazocine or fortral or lexir or talwin or phenoperidine or fenoperidine or lealgin or operidine or "r 1406" or r1406 or pirinitramid* or piritramid* or dipidolor or dipydolor or promedol or dimethylmeperidine or isopromedol or trimeperidine or sufentanil or sufentanilhameln or sulfentanil or sulfentanyl or "r 30730" or r30730 or tramadol or adolonta or amadol or biodalgic or biokanol or contramal or jutadol or "k 315" or k315 or mtwtramadol or nobligan or prontofort or ranitidin or takadol or theradol or tiral or topalgic or tradol or tradolpuren or tradonal or tralgiol or trama or tramadorsch or tramabeta or tramadin or tramadoc or tramadoldolgit or tramadolhameln or tramadolor or tramadolratiopharm or tramadura or tramagetic or tramagit or tramake or tramal or tramex or tramundin or trasedal or ultram or xymel or zamudol or zumalgic or zydol or zytram or acetorophine or acetylcodeine or acetymethadol or anileridine or apadoline or azidomorphine or benzhydrocodone or bezitramide or bremazocine or "brompton mixture" or ciramadol or cocomadol or codydramol or conorfone or cyclazocine or dextrorphan or dezocine or diamorphine or diconal or dihydroetorphine or dihydromorphine or dimethylthiambutene or dipipanone or dynorphin or enadoline or eptazocine or ethylketazocine or ethylmorphine or etonitazene or etorphine or etoxeridine or faxeladol or furethidine or gelonida or isalmadol or

isomethodone or ketazocine or ketobemidone or ketogan or kyotorphin or lefetamine or levacetylmethadol or levomethadone or levorphanol or metazocine or methylsamidorphan or tilidine or nicodine or nicomorphine or noracymethadol or bufigen or nubain* or nalbufin* or nalcryn or nalpain or onfor or noracymethadol or norbuprenorphine or normorphine or norpethidine or norpropoxyphene or nortramadol or oliceridine or oripavine or pentamorphone or phenadoxone or phencyclidine or picenadol or piminodine or piritramide or profadol or propiram or sameridine or samidorphan or semorphone or tapentadol or thebaine or tifluadom or tilidine or tonazocine or vicodin)

#3  #1 AND #2

#4  TS=(preoperativ* or "pre-operativ*" or "pre-surg*" or presurg* or ((prior or  "before") near/3 (surg* or arthroplasty or replacement)))

#5  #3 AND #4

**CINAHL Plus with Full-Text**

(MH "Arthroplasty+") or arthroplasty or (joint or knee* or hip* or shoulder*) n2 replacement

AND

( (MH "Analgesics, Opioid+") OR (MH "Narcotics+") ) OR ( opiate* or opioid* or narcotic* or morphin* or duramorph or "ms contin" or morphia or oramorph or "sdz 202 250" or sdz202250 or "sdz202 250" or alfenta or alfentanil or fanaxal or limifen or rapifen or "r 39209" or r39209 or alphaprodine or nisentil or prodine or buprenorphine or buprenex or buprex or prefin or subutex or temgesic or "6029 m" or 6029m or rx6029m or butorphanol or dolorex or moradol or stadol or torbugesic or "bc 2627" or bc2627 or codeine or ardinex or idocodeine or methylmorphine or dextromoramide or moramide or palfium or pyrrolamidol or dextropropoxyphene or propoxyphene or darvon or propoxyphene or enkephalin or dago or dagol or damge or damgo or "rx 783006" or rx783006 or dpdpe or fentanyl or duragesic or durogesic or fentanest or fentora or phentanyl or "r 4263" or r4263 or sublimaze or hydrocodon* or codinovo or dicodid or dihydrocodeinone or hycodan or hycon or hydrocodeinonebitartrate or robidone or hydromorphon* or dihydromorphinone or dilaudid or laudacon or palladone or meperidine or demerol or dolantin or dolargan or dolcontral or dolin or dolosal or dolsin or isonipecain or lidol or lydol or operidine or pethidine or meptazinol or meptid or "wy 22811" or wy22811 or nalbuphine or nubain or "en 2234a" or en2234a or oxycodone or dihydrohydroxycodeinone or dihydrone or dinarkon or eucodal or oxiconum or oxycodeinon or oxycone or oxycontin or pancodine or theocodin or percocet or oxymorphone or numorphan or opana or pentazocine or fortral or lexir or talwin or phenoperidine or fenoperidine or lealgin or operidine or "r 1406" or r1406 or pirinitramid* or piritramid* or dipidolor or dipydolor or promedol or dimethylmeperidine or isopromedol or trimeperidine or sufentanil or sufentanilhameln or sulfentanil or sulfentanyl or "r 30730" or r30730 or tramadol or adolonta or amadol or biodalgic or biokanol or contramal or jutadol or "k 315" or k315 or mtwtramadol or nobligan or prontofort or ranitidin or takadol or theradol or tiral or topalgic or tradol or tradolpuren or tradonal or tralgiol or trama or tramadorsch or tramabeta or tramadin or tramadoc or tramadoldolgit or tramadolhameln or tramadolor or tramadolratiopharm or tramadura or tramagetic or tramagit or tramake or tramal or tramex or tramundin or trasedal or ultram or xymel or zamudol or zumalgic or zydol or zytram or acetorophine or acetylcodeine or acetymethadol or anileridine or apadoline or azidomorphine or benzhydrocodone or bezitramide or bremazocine or "brompton mixture" or ciramadol or cocomadol or codydramol or conorfone or cyclazocine or dextrorphan or dezocine or diamorphine or diconal or dihydroetorphine or dihydromorphine or dimethylthiambutene or dipipanone or dynorphin or enadoline or eptazocine or ethylketazocine or ethylmorphine or etonitazene or etorphine or etoxeridine or faxeladol or furethidine or gelonida or isalmadol or  isomethodone or ketazocine or ketobemidone or ketogan or kyotorphin or lefetamine or levacetylmethadol or levomethadone or levorphanol or metazocine or methylsamidorphan or tilidine or nicodine or nicomorphine or noracymethadol or bufigen or nubain* or nalbufin* or nalcryn or nalpain or onfor or noracymethadol or norbuprenorphine or normorphine or norpethidine or norpropoxyphene or nortramadol or oliceridine or oripavine or pentamorphone or phenadoxone or phencyclidine or picenadol or piminodine or piritramide or profadol or propiram or sameridine or samidorphan or semorphone or tapentadol or thebaine or tifluadom or tilidine or tonazocine or vicodin )

AND

( (MH "Preoperative Care") OR (MH "Preoperative Period") ) OR ( preoperativ* or "pre-operativ*" or "pre-surg*" or presurg* ) or ((prior or  "before") w3 (surg* or arthroplasty or replacement))

**APPENDIX B:** **JBI Critical Appraisal Checklist for Cohort Studies**

Reviewer Date

Author Year Record Number

|  | Yes | No | Unclear | Not applicable |
| --- | --- | --- | --- | --- |
| 1. Were the two groups similar and recruited from the same population? | □ | □ | □ | □ |
| 1. Were the exposures measured similarly to assign people   to both exposed and unexposed groups? | □ | □ | □ | □ |
| 1. Was the exposure measured in a valid and reliable way? | □ | □ | □ | □ |
| 1. Were confounding factors identified? | □ | □ | □ | □ |
| 1. Were strategies to deal with confounding factors stated? | □ | □ | □ | □ |
| 1. Were the groups/participants free of the outcome at the start of the study (or at the moment of exposure)? | □ | □ | □ | □ |
| 1. Were the outcomes measured in a valid and reliable way? | □ | □ | □ | □ |
| 1. Was the follow up time reported and sufficient to be long enough for outcomes to occur? | □ | □ | □ | □ |
| 1. Was follow up complete, and if not, were the reasons to loss to follow up described and explored? | □ | □ | □ | □ |
| 1. Were strategies to address incomplete follow up utilized? | □ | □ | □ | □ |
| 1. Was appropriate statistical analysis used? | □ | □ | □ | □ |

Overall appraisal: Include □ Exclude □ Seek further info □

Comments (Including reason for exclusion)
