# Supplementary material for: Preoperative opioid use is associated with worse patient outcomes after Total joint arthroplasty: a systematic review and meta-analysis
Source: BMC Musculoskelet Disord. 2019 May 18;20:234. doi: 10.1186/s12891-019-2619-8 (PMC6525974; doi:10.1186/s12891-019-2619-8)
Supplement: Supplementary file 2 — Table S1. Additional Data provided for Goesling et at (2016). Table S2. Original Extracted Patient-Reported Outcome Scores. Table S3. Comparison of Secondary Outcomes between Patient Prescribed Preoperative Opioids and Opioid-Naïve Patients. (DOCX 29 kb) [file 12891_2019_2619_MOESM2_ESM.docx]

Additional file 2

**Table S1 – Additional Data provided for Goesling *et at* (2016)**

|  | **Pre-op opioid users** | | | | **Non-users** | | | |
| --- | --- | --- | --- | --- | --- | --- | --- | --- |
| **Score** | **n** | **Day of surgery** | **6 months** | **p value^1^** | **n** | **Day of surgery** | **6 months** | **p value^2^** |
| WOMAC Pain (SD) | 111 | 12.1 (3.2) | 3.6 (3.8) | <0.001 | 313 | 10.0 (3.3) | 2.4 (2.6) | <0.001 |
| WOMAC Stiffness (SD) | 111 | 5.1 (1.7) | 2.1 (1.7) | <0.001 | 315 | 4.4 (1.8) | 1.9 (1.6) | <0.001 |
| WOMAC Functioning (SD) | 108 | 41.0 (10.3) | 13.1 (11.9) | <0.001 | 308 | 34.2 (10.4) | 9.9 (9.2) | <0.001 |
| WOMAC Total (SD) | 104 | 58.3 (14.1) | 18.4 (16.6) | <0.001 | 306 | 48.6 (14.1) | 13.9 (12.3) | <0.001 |

**Abbreviations**

WOMAC - The Western Ontario and McMaster Universities Osteoarthritis Index, n number of patients.

**Notes**

^1^ Pairwise comparisons testing differences of scores at day of surgery and 6 months for pre-op opioid users

^2^ Pairwise comparisons testing differences of scores at day of surgery and 6 months for non-opioid users

**Table S2 – Original Extracted Patient-Reported Outcome Scores**

| **Study** | **Outcome Assessed** | **Statistic** | **Scoring** | **Preoperative Score** | | | **Post-operative Score** | | | **Change** | | | |
| --- | --- | --- | --- | --- | --- | --- | --- | --- | --- | --- | --- | --- | --- |
|  |  |  |  | **OU** | **nOU** | **p** | **OU** | **nOU** | **p** | **OU** | **p** | **nOU** | **p** |
| Zywiel *et al.* | KSS | mean (range) [95% CI] | 0 to 100 point-scale (100 indicates the best possible score) | 38 (11-63) [33, 42] | 37(10-55) [33, 41] | 0.513 | 79 (45 – 100) [76, 83] | 92 (59 - 100) [89 - 95] | <0.001 | 41 | - | 55 | - |
| Smith *et al.* | WOMAC Pain | mean [95% CI] | Transformed to a 0 to 100-point scale (100 indicating the worst possible score) | 44.6 [40.3, 48.9] | 43.7 [41.4, 46.0] | <0.05 | 17.1[12.8, 21.4] | 10.5 [8.3,12.8] | <0.05 | 27.0 [22.7, 31.3] | <0.05 | 33.6 [31.4, 35.9] | <0.05 |
| Franklin *et al.* | KSS | mean (SD) | 0 to 100 point-scale (100 indicates the best possible score) | 34.79 (15.17) | 37.06 (15.57) | <0.001 | 81.3^1^ (15.7) | 86^1^ (14.1) | - | 46.51 | - | 48.94 | - |
| Pivec *et al.* | HHS | mean (range) | 0 to 100-point scale (100 indicates the best score) | 43 | 45 | 0.26 | 84 (48 - 100) | 91 (74-100) | 0.002 | 41 | 0.01 | 49 | 0.01 |
| Nguyen *et al.* | WOMAC | mean | 0 to 100-point scale (100 indicates the best possible score) | 47.5 | 44.1 | - | 65.3 | 83.1 | <0.01 | 21.2 | - | 39 | - |
| Goesling *et al.* | WOMAC Pain, | mean (SD) | 0 - 20, higher indicates worse score | 12.1 (3.2) | 10.0 (3.3) | P<0.001 | 3.6 (3.8) | 2.4 (2.6) | - | 8.5 | <0.001 | 7.6 | <0.001 |
|  | WOMAC Function | mean (SD) | 0 - 68, higher indicates worse score | 41.0 (10.3) | 34.2 (10.4) | P<0.001 | 13.1 (11.9) | 9.9 (9.2) | - | 27.9 | <0.001 | 20.1 | <0.001 |
|  | WOMAC Stiffness | mean (SD) | 0 - 8, higher indicates wore score | 5.1 (1.7) | 4.4 (1.8) | P<0.001 | 2.1 (1.7) | 1.9 (1.6) | - | 3 | <0.001 | 2.3 | <0.001 |
|  | WOMAC Total | mean (SD) | 0 - 96, higher indicates worse | 58.3 (14.1) | 48.6 (14.1) | P<0.001 | 18.4 (16.6) | 13.9 (12.3) | - | 39.9 | <0.001 | 34.7 | <0.001 |

**Abbreviations**

PRO –Joint or Disease Specific Patient-Reported Outcome Score. All scores Transformed to a 0 to 100-point scale (100 indicating the best possible score), WOMAC – The Western Ontario and McMaster Universities Osteoarthritis Index, KSS – Knee Society Score, HHS – Harris Hip Score, OU – Preoperative opioid use, nOU – preoperative opioid-naïve, n – number of patients, SD – Standard deviation, CI – Confidence Interval, ‘-‘ indicates note reported in study

**Notes**

^1^OU and NonOU postoperative score calculated based on pooled stratified data extracted from paper

**Table S3 – Comparison of Secondary Outcomes between Patient Prescribed Preoperative Opioids and Opioid-Naïve Patients**

| **Study** | **Secondary Outcome** | **OU** | **nOU** | **p value** |
| --- | --- | --- | --- | --- |
| Zywiel *et al.* | Mean LOS (range) Mean morphine equivalence at discharge [95% CI]  Number of arthroscopic evaluations for unexplained pain [95% CI] Number of referrals to chronic pain clinic [95% CI] Number of revisions for recalcitrant pain and/or stiffness [95% CI] Mean ROM at final follow up [95% CI] | 4.3 (2-8)  85 mg [65, 106] 5 [2, 11] 10 [6, 17] 8 [6, 17] 107 [102, 113] | 3.4 [2-6] 91 mg [67, 115] 0 [0, 4] 1 [0, 6] 0 [0, 6] 111 [107, 114] | 0.013 0.946 0.066 <0.001 <0.001 0.223 |
| Franklin *et al.* | Opioid use at 12 months follow up^1^ | 14.0 | 2.6 | - |
| Pivec *et al.* | Mean LOS (range) Number of patients discharged to acute care rehabilitation facilities Mean morphine equivalences at 6 weeks follow up  Percent of patients using opioids at final follow up Number of Revisions | 4 (2 - 10) 30 63 mg 19% 2 | 3 (2 - 8) 24 2mg 4% 2 | 0.01 0.37 <0.001 0.04 - |
| Goesling *et al.* | Percent of TKA patients reporting opioids use at 1 months follow up Percent of TKA patients reporting opioids use at 2 months follow up Percent of TKA patients reporting opioids use at 6 months follow up  Percent of THA patients reporting opioids use at 1 months follow up Percent of THA patients reporting opioids use at 2 months follow up Percent of THA patients reporting opioids use at 6 months follow up | 88.5 48.2 53.3  63.9 37.8 34.7 | 66.5 16.6 8.2  22.5 4.4 4.3 | - - 0.001  - - <0.001 |

**Abbreviations**

CI – Confidence Interval, TKA – Total Knee Arthroplasty, THA – Total Hip Arthroplasty, OU – patients prescribed preoperative opioids, nOU – Preoperative opioid-naïve patients, ‘-‘ not reported in study

**Notes**

**^1^** 12% of preoperative opioid-naïve patients missing 12 months opioid data, 9% of preoperative opioid users missing 12 months opioid data
